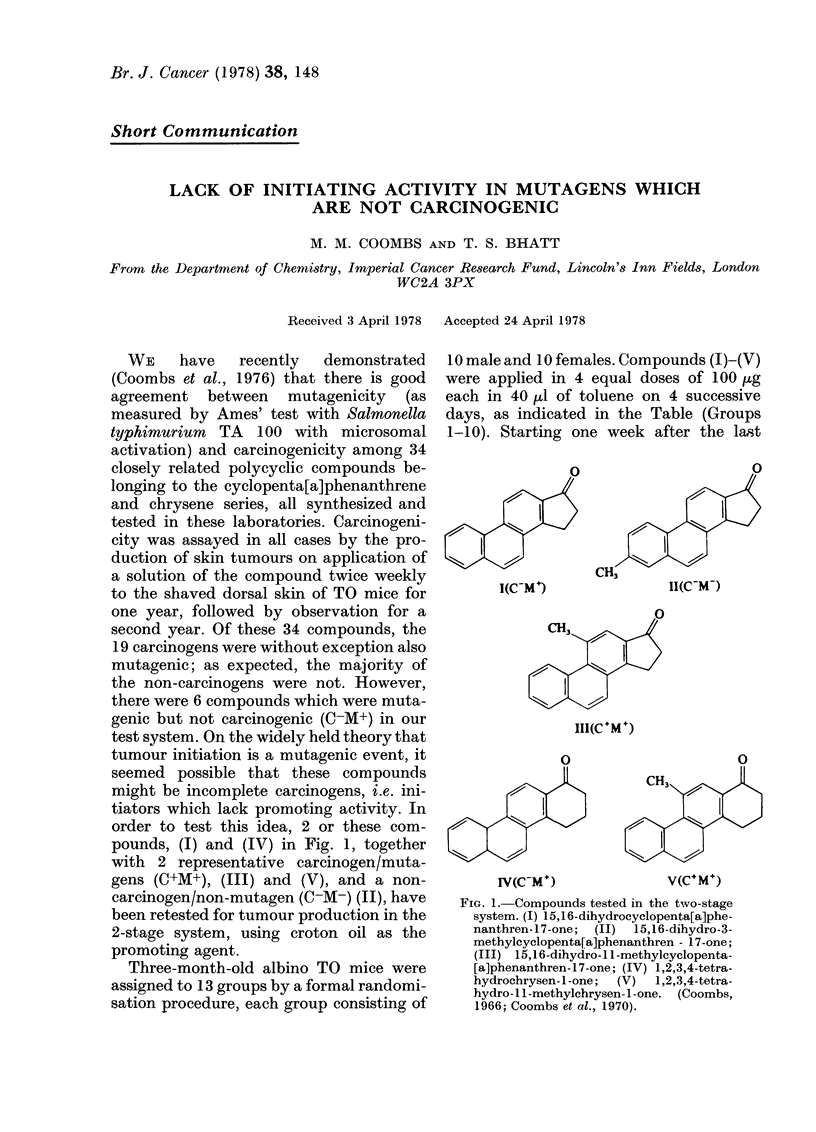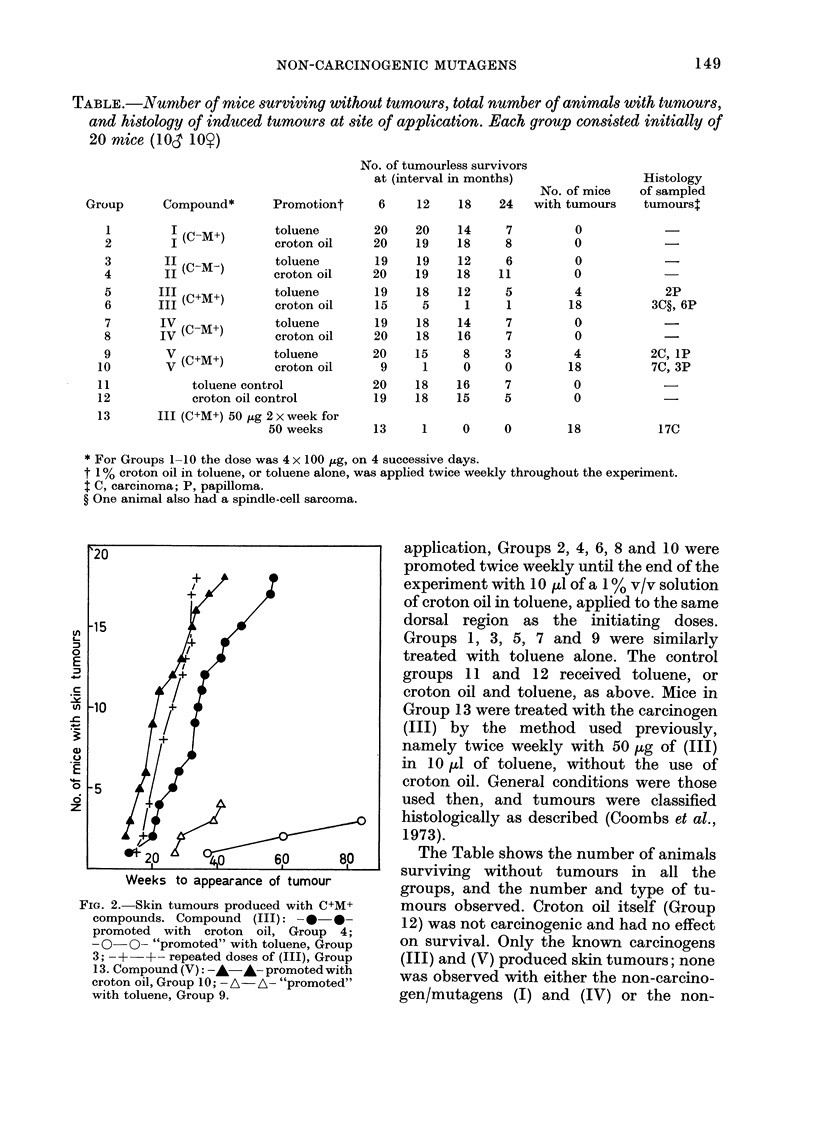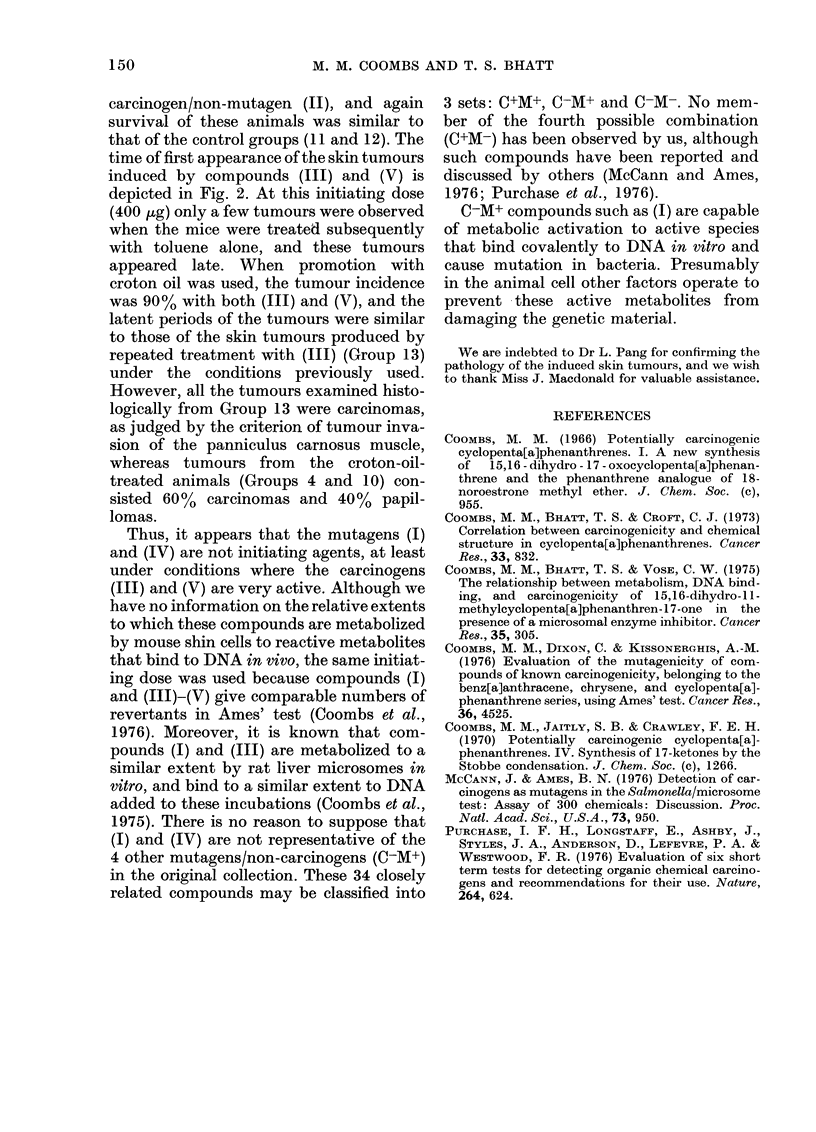# Lack of initiating activity in mutagens which are not carcinogenic.

**DOI:** 10.1038/bjc.1978.175

**Published:** 1978-07

**Authors:** M. M. Coombs, T. S. Bhatt


*Br. J. Cancer* (1978) **38**, 148

### *Short Communication*

### LACK OF INITIATING ACTIVITY IN MUTAGENS WHICH ARE NOT CARCINOGENIC

M. M. COOMBS AND T. S. BHATT

*From the Department of Chemistry, Imperial Cancer Research Fund, Lincoln's Inn Fields, London WC2A 3PX*

Received 3 April 1978 Accepted 24 April 1978

WE have recently demonstrated (Coombs *et al.*, 1976) that there is good agreement between mutagenicity (as measured by Ames' test with *Salmonella typhimurium* TA 100 with microsomal activation) and carcinogenicity among 34 closely related polycyclic compounds belonging to the cyclopenta[a]phenanthrene and chrysene series, all synthesized and tested in these laboratories. Carcinogenicity was assayed in all cases by the production of skin tumours on application of a solution of the compound twice weekly to the shaved dorsal skin of TO mice for one year, followed by observation for a second year. Of these 34 compounds, the 19 carcinogens were without exception also mutagenic; as expected, the majority of the non-carcinogens were not. However, there were 6 compounds which were mutagenic but not carcinogenic ($C^-M^+$) in our test system. On the widely held theory that tumour initiation is a mutagenic event, it seemed possible that these compounds might be incomplete carcinogens, *i.e.* initiators which lack promoting activity. In order to test this idea, 2 or these compounds, (I) and (IV) in Fig. 1, together with 2 representative carcinogen/mutagens ($C^+M^+$), (III) and (V), and a non-carcinogen/non-mutagen ($C^-M^-$) (II), have been retested for tumour production in the 2-stage system, using croton oil as the promoting agent.

Three-month-old albino TO mice were assigned to 13 groups by a formal randomisation procedure, each group consisting of 10 male and 10 females. Compounds (I)–(V) were applied in 4 equal doses of 100 $\mu$g each in 40 $\mu$l of toluene on 4 successive days, as indicated in the Table (Groups 1–10). Starting one week after the last

I($C^-M^+$)

II($C^-M^-$)

III($C^+M^+$)

IV($C^-M^+$)

V($C^+M^+$)

FIG. 1.—Compounds tested in the two-stage system. (I) 15,16-dihydrocyclopenta[a]phenanthren-17-one; (II) 15,16-dihydro-3-methylcyclopenta[a]phenanthren - 17-one; (III) 15,16-dihydro-11-methylcyclopenta-[a]phenanthren-17-one; (IV) 1,2,3,4-tetrahydrochrysen-1-one; (V) 1,2,3,4-tetrahydro-11-methylchrysen-1-one. (Coombs, 1966; Coombs *et al.*, 1970).

TABLE.—*Number of mice surviving without tumours, total number of animals with tumours, and histology of induced tumours at site of application. Each group consisted initially of 20 mice* (10♂ 10♀)

| Group | Compound* | Promotion† | No. of tumourless survivors at (interval in months) 6 | 12 | 18 | 24 | No. of mice with tumours | Histology of sampled tumours‡ |
|---|---|---|---|---|---|---|---|---|
| 1 | I ($C^-M^+$) | toluene | 20 | 20 | 14 | 7 | 0 | — |
| 2 | I ($C^-M^+$) | croton oil | 20 | 19 | 18 | 8 | 0 | — |
| 3 | II ($C^-M^-$) | toluene | 19 | 19 | 12 | 6 | 0 | — |
| 4 | II ($C^-M^-$) | croton oil | 20 | 19 | 18 | 11 | 0 | — |
| 5 | III ($C^+M^+$) | toluene | 19 | 18 | 12 | 5 | 4 | 2P |
| 6 | III ($C^+M^+$) | croton oil | 15 | 5 | 1 | 1 | 18 | 3C§, 6P |
| 7 | IV ($C^-M^+$) | toluene | 19 | 18 | 14 | 7 | 0 | — |
| 8 | IV ($C^-M^+$) | croton oil | 20 | 18 | 16 | 7 | 0 | — |
| 9 | V ($C^+M^+$) | toluene | 20 | 15 | 8 | 3 | 4 | 2C, 1P |
| 10 | V ($C^+M^+$) | croton oil | 9 | 1 | 0 | 0 | 18 | 7C, 3P |
| 11 | toluene control | | 20 | 18 | 16 | 7 | 0 | — |
| 12 | croton oil control | | 19 | 18 | 15 | 5 | 0 | — |
| 13 | III ($C^+M^+$) 50 μg 2 × week for 50 weeks | | 13 | 1 | 0 | 0 | 18 | 17C |

* For Groups 1–10 the dose was 4 × 100 μg, on 4 successive days.
† 1% croton oil in toluene, or toluene alone, was applied twice weekly throughout the experiment.
‡ C, carcinoma; P, papilloma.
§ One animal also had a spindle-cell sarcoma.

FIG. 2.—Skin tumours produced with $C^+M^+$ compounds. Compound (III): –●—●– promoted with croton oil, Group 4; –○—○– "promoted" with toluene, Group 3; –+—+– repeated doses of (III), Group 13. Compound (V): –▲—▲– promoted with croton oil, Group 10; –△—△– "promoted" with toluene, Group 9.

application, Groups 2, 4, 6, 8 and 10 were promoted twice weekly until the end of the experiment with 10 μl of a 1% v/v solution of croton oil in toluene, applied to the same dorsal region as the initiating doses. Groups 1, 3, 5, 7 and 9 were similarly treated with toluene alone. The control groups 11 and 12 received toluene, or croton oil and toluene, as above. Mice in Group 13 were treated with the carcinogen (III) by the method used previously, namely twice weekly with 50 μg of (III) in 10 μl of toluene, without the use of croton oil. General conditions were those used then, and tumours were classified histologically as described (Coombs *et al.*, 1973).

The Table shows the number of animals surviving without tumours in all the groups, and the number and type of tumours observed. Croton oil itself (Group 12) was not carcinogenic and had no effect on survival. Only the known carcinogens (III) and (V) produced skin tumours; none was observed with either the non-carcinogen/mutagens (I) and (IV) or the noncarcinogen/non-mutagen (II), and again survival of these animals was similar to that of the control groups (11 and 12). The time of first appearance of the skin tumours induced by compounds (III) and (V) is depicted in Fig. 2. At this initiating dose (400 $\mu$g) only a few tumours were observed when the mice were treated subsequently with toluene alone, and these tumours appeared late. When promotion with croton oil was used, the tumour incidence was 90% with both (III) and (V), and the latent periods of the tumours were similar to those of the skin tumours produced by repeated treatment with (III) (Group 13) under the conditions previously used. However, all the tumours examined histologically from Group 13 were carcinomas, as judged by the criterion of tumour invasion of the panniculus carnosus muscle, whereas tumours from the croton-oil-treated animals (Groups 4 and 10) consisted 60% carcinomas and 40% papillomas.

Thus, it appears that the mutagens (I) and (IV) are not initiating agents, at least under conditions where the carcinogens (III) and (V) are very active. Although we have no information on the relative extents to which these compounds are metabolized by mouse shin cells to reactive metabolites that bind to DNA *in vivo*, the same initiating dose was used because compounds (I) and (III)–(V) give comparable numbers of revertants in Ames' test (Coombs *et al.*, 1976). Moreover, it is known that compounds (I) and (III) are metabolized to a similar extent by rat liver microsomes *in vitro*, and bind to a similar extent to DNA added to these incubations (Coombs *et al.*, 1975). There is no reason to suppose that (I) and (IV) are not representative of the 4 other mutagens/non-carcinogens ($C^-M^+$) in the original collection. These 34 closely related compounds may be classified into 3 sets: $C^+M^+$, $C^-M^+$ and $C^-M^-$. No member of the fourth possible combination ($C^+M^-$) has been observed by us, although such compounds have been reported and discussed by others (McCann and Ames, 1976; Purchase *et al.*, 1976).

$C^-M^+$ compounds such as (I) are capable of metabolic activation to active species that bind covalently to DNA *in vitro* and cause mutation in bacteria. Presumably in the animal cell other factors operate to prevent these active metabolites from damaging the genetic material.

We are indebted to Dr L. Pang for confirming the pathology of the induced skin tumours, and we wish to thank Miss J. Macdonald for valuable assistance.

### REFERENCES

Coombs, M. M. (1966) Potentially carcinogenic cyclopenta[a]phenanthrenes. I. A new synthesis of 15,16-dihydro-17-oxocyclopenta[a]phenanthrene and the phenanthrene analogue of 18-noroestrone methyl ether. *J. Chem. Soc.* (c), 955.

Coombs, M. M., Bhatt, T. S. & Croft, C. J. (1973) Correlation between carcinogenicity and chemical structure in cyclopenta[a]phenanthrenes. *Cancer Res.*, **33**, 832.

Coombs, M. M., Bhatt, T. S. & Vose, C. W. (1975) The relationship between metabolism, DNA binding, and carcinogenicity of 15,16-dihydro-11-methylcyclopenta[a]phenanthren-17-one in the presence of a microsomal enzyme inhibitor. *Cancer Res.*, **35**, 305.

Coombs, M. M., Dixon, C. & Kissonerghis, A.-M. (1976) Evaluation of the mutagenicity of compounds of known carcinogenicity, belonging to the benz[a]anthracene, chrysene, and cyclopenta[a]-phenanthrene series, using Ames' test. *Cancer Res.*, **36**, 4525.

Coombs, M. M., Jaitly, S. B. & Crawley, F. E. H. (1970) Potentially carcinogenic cyclopenta[a]-phenanthrenes. IV. Synthesis of 17-ketones by the Stobbe condensation. *J. Chem. Soc.* (c), 1266.

McCann, J. & Ames, B. N. (1976) Detection of carcinogens as mutagens in the *Salmonella*/microsome test: Assay of 300 chemicals: Discussion. *Proc. Natl. Acad. Sci., U.S.A.*, **73**, 950.

Purchase, I. F. H., Longstaff, E., Ashby, J., Styles, J. A., Anderson, D., Lefevre, P. A. & Westwood, F. R. (1976) Evaluation of six short term tests for detecting organic chemical carcinogens and recommendations for their use. *Nature*, **264**, 624.